# Serum level of (interleukin (IL)-2, IL-10, tumor necrosis factor (TNF)-a, motilin (MTL), gastrin (GAS), pepsinogen (PG), after adjunctive treatment in patients with chronic atrophic gastritis

**DOI:** 10.5937/jomb0-56027

**Published:** 2025-10-28

**Authors:** Pan Xue

**Affiliations:** 1 Xi'an Daxing Hospital, Geriatrics/Traditional Chinese Medicine Five Disciplines, Xi'an, Shaanxi Province, China

**Keywords:** serum level of (interleukin (IL)-2, IL-10, tumour necrosis factor (TNF)-a, motilin (MTL), gastrin (GAS), pepsinogen (PG), matrine, omeprazole enteric-coated tablets, chronic atrophic gastritis, Helicobacter pylori eradication rate, gastric mucosal histopathology, nivo u serumu (interleukin (IL)-2, IL-10, faktor nekroze tumora-alfa (TNF-a, motilin (MTL), gastrin (GAS), pepsinogen (PG), matrin, enterično obložene tablete omeprazola, hronični atrofični gastritis, stopa eradikacije Helicobacter pylori, histopatologija želudačne sluzokože

## Abstract

**Background:**

The study aimed to demonstrate the impact of traditional Chinese medicine (TCM) matrine combined with omeprazole enteric-coated tablets on gastric mucosal histopathology, gastric function, inflammatory cytokines, and Helicobacter pylori (H. pylori) eradication in patients with chronic atrophic gastritis (CAG).

**Methods:**

A retrospective collection of case data from 110 patients with CAG admitted to the TCM Department of the hospital was conducted. Patients were rolled into the test group (TG, matrine + omeprazole enteric-coated tablets) and the control group (CG, omeprazole enteric-coated tablets). The gastric mucosal histopathological scores, serological indicators (interleukin (IL)-2, IL-10, tumour necrosis factor (TNF-a), gastric function (motilin (MTL), gastrin (GAS), pepsinogen (PG)), H. pylori eradication rate, and clinical efficacy were compared.

**Results:**

The degree of glandular atrophy, intestinal meta-plasia, dysplasia, and inflammatory activity index in patients of TG post-treatment were lower than CG (P&lt;0.05). Post-treatment, IL-2, IL-10, and TNF-a levels in patients of TG were inferior to CG (P&lt;0.05), and the H. pylori eradication rate in patients of TG (87.27%) was inferior to CG (63.64%) (P&lt;0.05). The effective treatment rate in patients of TG post-treatment (92.73%) was higher than CG (78.18%) (P&lt;0.05).

**Conclusions:**

Matrine combined with omeprazole enteric-coated tablets significantly improved gastric mucosal histopathology, reduced inflammatory cytokine levels, enhanced gastric function, and increased the H. pylori eradication rate compared to omeprazole monotherapy.

## Abstract (sr)

**Uvod:**

Cilj ove studije bio je da pokaže uticaj matrina iz tradicionalne kineske medicine (TCM) u kombinaciji sa enterično obloženim tabletama omeprazola na histopatologiju želudačne sluzokože, funkciju žluca, inflamatorne citokine i eradikaciju Helicobacter pylori (H. pylori) kod pacijenata sa hroničnim atrofičnim gastritisom (CAG).

**Metode:**

Retrospektivno su prikupljeni podaci o slučajevima 110 pacijenata sa CAG, koji su bili hospitalizovani na Odeljenju za TKM. Pacijenti su podeljeni u ispitivanu grupu (TG, matrin + enterično obložene tablete omeprazola) i kontrolnu grupu (CG, omeprazol, enterično obložene tablete). Upoređivani su histopatološki skorovi želudačne sluzokože, serološki indikatori (interleukin (IL)-2, IL-10, faktor nekroze tumora-alfa (TNF-a), funkcija želuca (motilin (MTL), gastrin (GAS), pepsinogen (PG)), stopa eradikacije H. pylori i klinička efikasnost.

**Rezultati:**

Nakon tretmana, stepen atrofije žlezda, crevne metaplazije, displazije i indeks inflamatorne aktivnosti kod pacijenata TG bio je niži nego kod pacijenata CG (P&lt;0,05). Posle tretmana, nivoi IL-2, IL-10 i TNF-a kod pacijenata TG bili su niži nego kod pacijenata CG (P&lt;0,05), dok je stopa eradikacije H. pylori u IG (87,27%) bila viša nego u CG (63,64%) (P&lt;0,05). Efikasnost tret-mana kod pacijenata CG nakon tretmana (92,73%) je bila viša nego kod pacijenata CG (78,18%) (P&lt;0,05).

**Zaključak:**

Matrin u kombinaciji sa enterično obloženim tabletama omeprazola značajno poboljšava histopatologiju želudačne sluzokože, smanjuje nivoe inflamatornih citokina, poboljšava funkciju želuca i povećava stopu eradikacije H. pylori u poređenju sa monoterapijom omeprazolom.

## Introduction

Chronic atrophic gastritis (CAG) is a prevalent gastric disorder, typically arising from prolonged inflammation of the gastric mucosa. Its main pathological features include gastric mucosal atrophy, reduced glandular density, and decreased mucous and acid secretion [1] [2] [3]. This condition is often associated with Helicobacter pylori (H. pylori) infection, prolonged use of nonsteroidal anti-inflammatory drugs (aspirin and ibuprofen), or other gastric disorders (autoimmune diseases) [4]. Patients may present various digestive symptoms, including gastric pain, bloating, nausea, vomiting, heartburn, and loss of appetite. Endoscopic examination reveals changes in gastric mucosa, such as decreased smoothness, erythema, ulcers, and atrophy. Pathological examination typically shows glandular atrophy and degeneration [5] [6]. Common treatment modalities include anti-H. pylori therapy, gastric acid suppression, anti-inflammatory treatment, and nutritional support. However, conventional treatments such as gastric acid suppressants and antibiotic therapy, while partially alleviating symptoms, do not provide a complete cure for the disease, and some patients may exhibit drug resistance or adverse reactions [7]. Hence, a growing interest is in exploring new treatment strategies and drugs to address this clinical challenge.

Chinese herbal medicine adjunctive therapy integrates traditional Chinese medicine (TCM) with modern medical treatment methodologies to enhance therapeutic efficacy, reduce side effects, or enhance patients’ quality of life. This treatment approach has been widely applied in clinical practice and is gradually gaining attention and recognition [8] [9]. Zhang et al. [10] used a combination of Chinese herbal compound and Sanqi powder to treat patients with CAG erosions, compared with aluminium-magnesium suspension, revealing that the overall clinical efficacy of the Chinese herbal compound combined with Sanqi powder based on the comprehensive syndrome differentiation of TCM was superior to acid suppressants. Chen et al. [11] investigated the efficacy of QiruiGaishu capsules compared with the positive herb SanjiuWeitai capsules in treating subjects with chronic non-atrophic gastritis. They found that QiruiGaishu capsules seemed more effec tive in relieving symptoms than SanjiuWeitai capsules, with better alleviating upper abdominal pain and TCM symptoms. Matrine is a pharmaceutical preparation where matrines are dissolved in a suitable solvent and administered in injectable form. Matrine appears as a transparent or slightly yellow liquid and is commonly utilised in clinical practice [12] [13] [14]. The main active ingredient of this injection, matrine, is a type of bioalkaloid with various pharmacological effects, including anti-inflammatory, antibacterial, and antioxidant properties. In clinical application, matrine is commonly used to treat digestive system disorders, particularly inflammation-associated, such as chronic gastritis and gastric ulcers. Its mechanism of action primarily involves inhibiting inflammatory responses, tissue damage mitigation, and tissue repair promotion [15]. Matrine is often employed as an adjunctive therapy, combined with other drugs, to enhance therapeutic efficacy [16].

However, despite demonstrating particular therapeutic efficacy in clinical settings, the specific treatment mechanisms and dose-response effects of matrine still require further in-depth investigation. Research on the pharmacological actions, safety profile, and optimal dosing of matrine is paramount for expanding its scope in clinical applications and enhancing therapeutic outcomes. Our study aims to evaluate the clinical efficacy and safety of combining matrine with omeprazole in patients with CAG. By investigating this combination therapy, we seek to determine whether matrine can enhance treatment outcomes, reduce reliance on long-term PPI use, and ultimately improve patient quality of life. The findings could offer valuable insights into more effective and safer therapeutic strategies for managing chronic atrophic gastritis.

## Materials and methods

### Research object

In this retrospective cohort study, data from 110 patients diagnosed with CAG admitted to the TCM Department of Xi’an Daxing Hospital were collected by census method sampling from January 2023 to February 2024. The patients’ ages ranged from 45 to 75 years, with 58 males and 52 females, and disease durations ranging from 8 months to 15 years.

Diagnostic criteria: i) conforming to the diagnostic criteria outlined in the AGA Clinical Practice Update on the Diagnosis and Management of Atrophic Gastritis: Expert Review [17]; ii) endoscopic examination criteria for CAG: irregular mucosal appearance, pale colour, shallow gastric mucosal folds, and reduced or complete loss of glands; iii) TCM syndrome differentiation diagnostic criteria as shown in Table 1.

**Table 1:** Diagnostic criteria for TCM syndrome differentiation [18] [19].

Pattern identification	Characteristics	Tongue appearance	Pulse characteristics
Qi deficiency	Loss of appetite, fullness and oppression in the epigastrium, fatigue and lethargy	Pale with a thin white coating	Weak
Yin deficiency	Dry mouth, bitter taste in the mouth, dry throat, internal heat disturbing	Red or less moist with a thin yellow coating	Thin and weak
Damp-heat	Bitter taste in the mouth, bad breath, nausea, abdominal distension	Red with thick and greasy yellow coating	Slippery and weak

Inclusion criteria: i) age 18 years or older; ii) no relevant drug treatment in the previous month; iii) no history of drug allergies; iv) TCM syndrome differentiation diagnosis indicating deficiency of stomach yin; v) good treatment compliance.

Exclusion criteria: i) patients allergic to the drugs used in this trial; ii) severe gastric mucosal dysplasia; iii) patients with severe cardiovascular or pulmonary organ disorders; iv) severe psychiatric disorders; v) concomitant peptic ulcer disease.

### Grouping and treatment plan

The patients were rolled into a test group (TG) and a control group (CG) based on different treatment regimens, with 55 cases in each group. In TG, there were 30 male and 25 female patients, with an average age of 49.55±7.62 years and a disease duration of 4.72±1.85 years. There were 28 male and 27 female patients in CG, with an average age of 51.38±5.44 years and a disease duration of 5.05±1.49 years. The gender distribution, age, and disease duration differed slightly between the two groups (P>0.05).

The medication regimen for CG consisted of 20 mg omeprazole enteric-coated tablets (produced by Shanxi Yunpeng Pharmaceutical Group Co., Ltd., China, National Medical Products Administration approval number H20123239), taken orally, one tablet twice daily for one month. The medication regimen for TG involved adding matrine 0.15 g (produced by Shanxi Zhendong Pharmaceutical Co., Ltd., China, National Medical Products Administration approval number H20051310) to the treatment of CG. The injection was administered intravenously, with each dose of 150 mg (15 mL) diluted in 500 mL of 10% glucose injection solution and administered at approximately 60 drops per minute. The treatment was administered once daily for two weeks.

### Efficacy criteria

Table 2 shows patients’ clinical efficacy criteria [17], which includes four items: recovery, remarkably effective, effective, and ineffective.

**Table 2:** Clinical efficacy criteria for patients.

Classification of therapeutic effects	Symptoms improvement
Recovery	Complete eradication of CAG in patients, with resolution of gastric mucosal inflammation and complete relief of symptoms, resulting in restoration to a healthy state.
Remarkably effective	Treatment remarkably improved symptoms and inflammation in patients, leading to notable symptom reduction and improvement in inflammation, although complete resolution may not be achieved.
Effective	Treatment demonstrated clinical efficacy by alleviating inflammation, relieving symptoms, or preventing and reducing complications.
Ineffective	Treatment failed to produce the expected clinical outcomes, with no reduc- tion in inflammation or improvement in symptoms, and may even result in new complications or worsening of symptoms.

### Therapeutic efficacy indicators

Gastric mucosal histopathological scores (0 points indicating no abnormality or normal; 1 point: mild abnormality or slight lesion; 2 points: moderate abnormality or moderate lesion; 3 points: severe abnormality or severe lesion) and TCM syndrome scores (including epigastric distension, epigastric pain, decreased appetite, heartburn, acid reflux) pre- and post-treatment were collected in both groups of patients.

Serum markers pre- and post-treatment were collected in both groups of patients, including interleukin (IL)-2, IL-10, tumour necrosis factor (TNF)-α, motilin (MTL), gastrin (GAS), and pepsinogen (PG).

The H. pylori eradication rate post-treatment was collected (the detection method involved collecting fasting breath samples from patients using a carbon-13 breath analyser (Beijing Safe Heart Intelligent Technology Co., Ltd., China), followed by analysis of H. pylori infection status using the MAT253 stable isotope mass spectrometer (ThermoFisher, USA)).

### Statistical methodologies

Using SPSS 19.0, continuous variables were indicated as mean±standard deviation, while categorical data were shown as percentages (%). Between-group comparisons were conducted utilising repeated measures analysis of variance (ANOVA), and within-group comparisons were performed using two-way ANOVA. Two-tailed tests were employed, with P<0.05 indicating statistical significance.

## Results

### TCM syndrome scores

**Figure 1:**
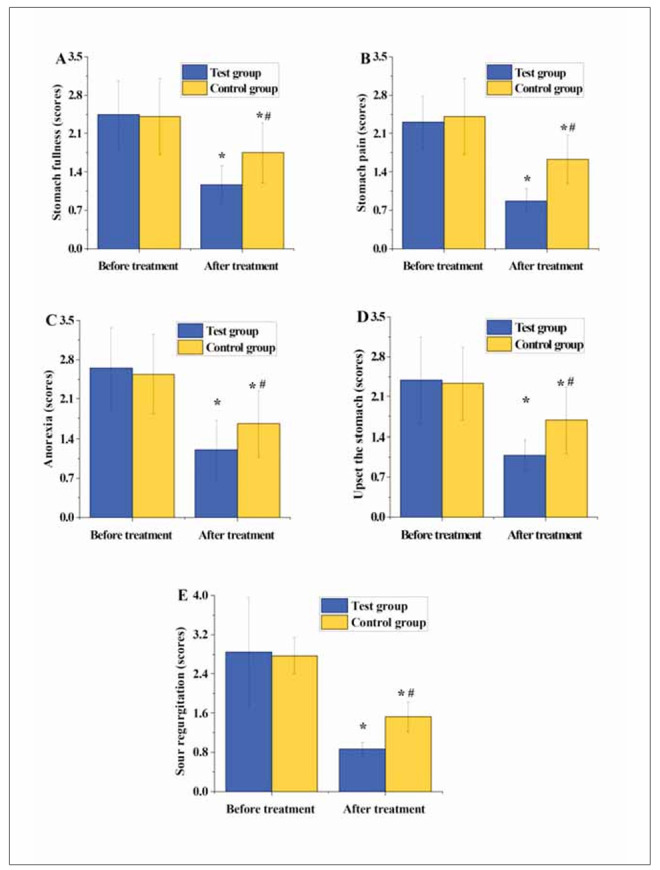
Preoperative CK-MB levels of patients.

In Figure 1, the scores for epigastric distension, epigastric pain, decreased appetite, heartburn, and acid reflux in both groups of patients markedly decreased post-treatment versus pre-treatment (P<0.05). Additionally, the scores for epigastric distension, epigastric pain, decreased appetite, heartburn, and acid reflux in TG were notably inferior to those in CG post-treatment (P<0.05).

### Comparison of gastric mucosal histopathological scores between two groups of patients

**Figure 2:**
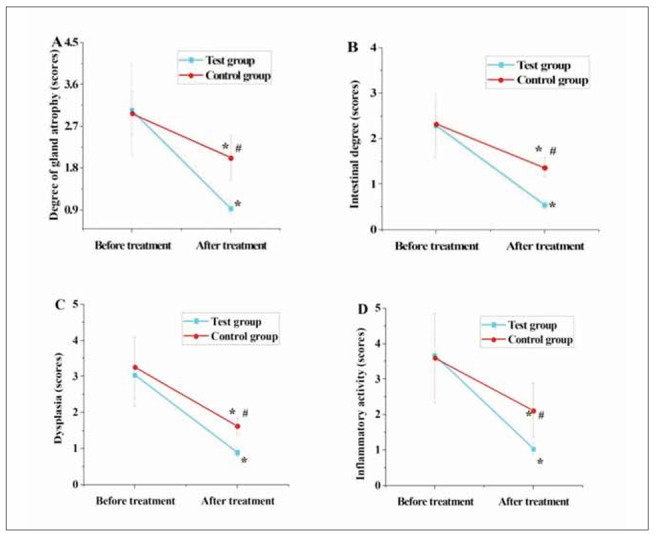
The comparison of gastric mucosal histopathological scores between the two groups of patients.<br>(A–D represent glandular atrophy, intestinal metaplasia, dysplasia, and inflammatory activity, respectively)

In Figure 2, the scores for glandular atrophy, intestinal metaplasia, dysplasia, and inflammatory activity in both groups of patients significantly decreased post-treatment relative to pre-treatment (P<0.05). Furthermore, the scores for glandular atrophy, intestinal metaplasia, dysplasia, and inflammatory activity in TG were substantially inferior to those in CG post-treatment (P<0.05).

### Serum inflammatory factor levels pre- and post-treatment

**Figure 3:**
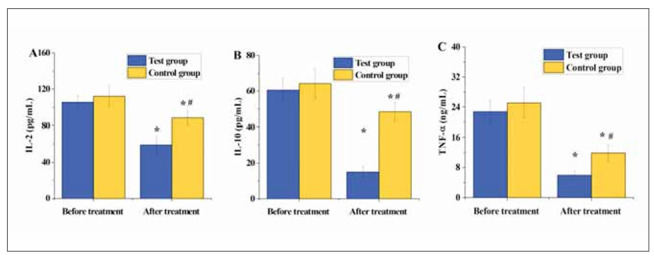
The comparison of gastric mucosal histopathological scores between the two groups of patients. (A–D represent glandular atrophy, intestinal metaplasia, dysplasia, and inflammatory activity, respectively)

In Figure 3, following treatment, IL-2, IL-10, and TNF-α in both patient groups were significantly lower versus pre-treatment levels, with considerable differences observed (P<0.05). Furthermore, IL-2, IL-10, and TNF-α post-treatment in TG were significantly inferior to CG, with marked differences observed (P<0.05).

### Comparison of gastric function indicators pre- and post-treatment

**Figure 4:**
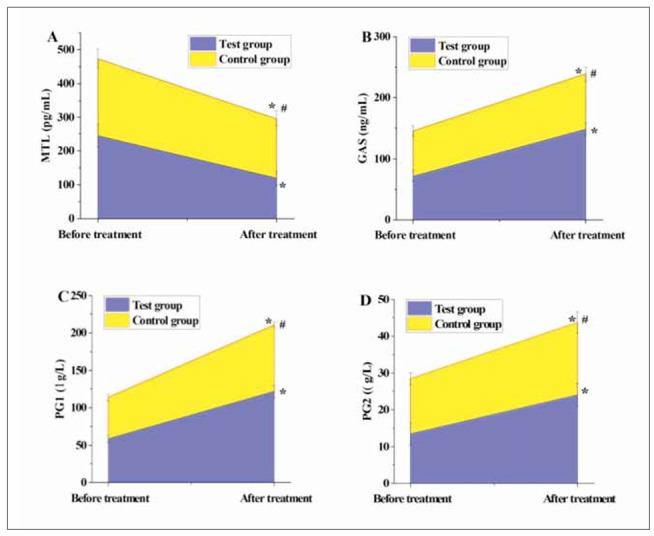
The comparison of the gastric function indicators.

In Figure 4, following treatment, the levels of MTL decreased drastically relative to pre-treatment levels, while the levels of GAS, PG , and PG significantly increased versus pre-treatment levels in both groups of patients (P<0.05). Moreover, post-treatment MTL levels in TG were markedly inferior to those in CG, while GAS, PG , and PG levels were significantly higher in TG versus CG (P<0.05).

### Negative conversion of H. pylori in two groups of patients post-treatment

**Figure 5:**
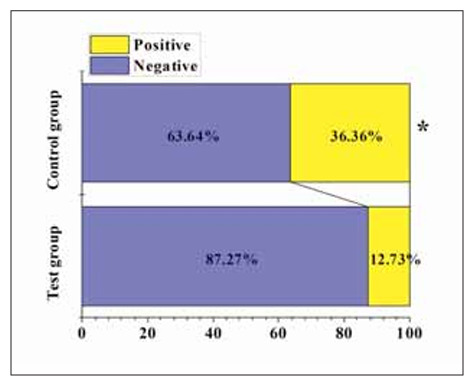
The post-treatment H. pylori clearance status in two groups of patients.

In Figure 5, 48 cases tested negative for H. pylori post-treatment among patients in TG, resulting in a conversion rate of 87.27%. In contrast, among patients in CG, 35 cases tested negative for H. pylori post-treatment, resulting in a conversion rate of 63.64%. It was observed that the conversion rate of H. pylori post-treatment in TG was markedly inferior to that in CG (P<0.05).

### Comparison of clinical efficacy between two groups of patients

**Figure 6:**
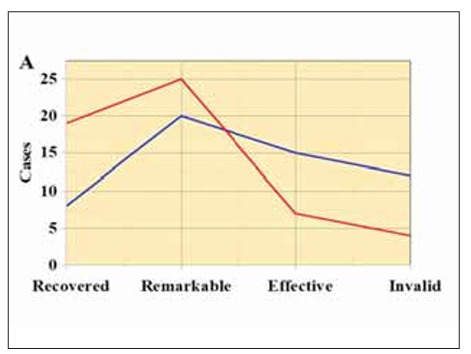
The comparison of the clinical efficacy between the two groups of patients.<br>(A displays the number of cases for each category: complete recovery, remarkable improvement, effective, and ineffective; B presents the treatment effectiveness rate)

In Figure 6, among patients in TG, 19 cases achieved complete recovery, 25 cases showed remarkable improvement, 7 cases were classified as effective, and 4 cases were deemed ineffective. In comparison, among patients in CG, 8 cases achieved complete recovery, 20 cases showed remarkable improvement, 15 were classified as effective, and 12 were deemed ineffective. The treatment effectiveness rate in TG (92.73%) was dramatically higher than that in CG (78.18%) (P<0.05).

## Discussion

Traditional Chinese medicine refers to herbal therapy originating from ancient China and evolving throughout history. It often consists of natural substances such as plants, animals, and minerals, prepared into medicinal formulations through decoction, infusion, and grinding. It treats diseases and maintains health [18] [19]. Matrine is a natural compound primarily found in the herb Sophora flavescens, also known as Kushen or Sophora root. It belongs to the class of alkaloids and exhibits various pharmacological effects, which are widely utilised in traditional Chinese medicine. Its main functions include antimicrobial, antiviral, antitumor, anti-inflammatory, and antioxidative properties [20] [21] [22]. Consequently, matrine is employed in TCM to treat various ailments such as infections, inflammations, and tumours [23] [24]. Therefore, this study retrospectively collected medical records of 110 patients with CAG enrolled in the TCM Department of a hospital. Based on different treatment regimens, patients were divided into a TG (matrines + omeprazole enteric-coated tablets) and a CG (omeprazole enteric-coated tablets), each comprising 55 cases. The study compared the pre- and post-treatment gastric mucosal histopathological scores, serological indicators (IL-2, IL-10, TNF-α), gastric function (MTL, GAS, PG), and clinical efficacy between the two groups.

Our study investigated the therapeutic effects of matrine combined with omeprazole enteric-coated tablets on chronic atrophic gastritis. The combination therapy significantly improved gastric mucosal histopathology, reduced inflammatory cytokine levels, enhanced gastric function, and increased the Helicobacter pylori eradication rate compared to omeprazole monotherapy. These findings suggest that matrine, with its anti-inflammatory and antioxidative properties, can enhance the efficacy of standard treatment for chronic atrophic gastritis, offering a promising complementary approach in clinical practice.

Our results align with previous research indicating the beneficial effects of matrine on gastrointestinal health. For instance, matrine has been shown to exert protective effects against gastric mucosal injury by modulating inflammatory responses and oxidative stress [25]. Furthermore, its antimicrobial properties may contribute to eradicating H. pylori, a common pathogen associated with chronic atrophic gastritis [22].

Comparing the scores of TCM syndromes assists TCM practitioners in systematically assessing the patient’s condition and underlying pathogenesis, thus providing a basis for developing personalised treatment plans. In TCM diagnosis, based on the patient’s symptoms, tongue diagnosis, pulse diagnosis, and other information, combined with TCM theory, the disease is categorised into different syndromes, each with its specific pathogenesis and treatment methods [26] [27]. This study found that post-treatment, patients in TG exhibited notably lower scores for symptoms such as epigastric distention, epigastric pain, decreased appetite, heartburn, and acid reflux compared to CG (P<0.05). This suggests that the adjunctive use of matrines with omeprazole enteric-coated tablets can effectively alleviate gastric-related symptoms in patients, yielding better results than a single Western medicine treatment [28]. Additionally, the degree of glandular atrophy, intestinal metaplasia, dysplasia, and inflammatory activity scores were significantly lower in TG than in CG (P<0.05). This finding is similar to the results of a study on the treatment of H. pylori-related chronic gastritis using quadruple therapy containing bismuth by Wang et al. [29], indicating that the adjunctive use of matrines with omeprazole enteric-coated tablets can effectively alleviate symptoms of gastric mucosal tissue in patients.

The abnormal expression or sustained release of inflammatory factors may lead to a chronic inflammatory state, increasing the risk of cardiovascular diseases, tumours, autoimmune diseases, and other conditions [30] [31]. This study found that post-treatment IL-2, IL-10, and TNF-α levels in patients of TG were significantly inferior to CG (P<0.05). This indicates that the adjunctive use of matrines with omeprazole enteric-coated tablets can effectively reduce the levels of inflammatory factors in patients, thus inhibiting the progression of chronic inflam mation. Additionally, Tan et al. [32] utilised standarddose zinc-l-carnosine combined with triple therapy (omeprazole 20 mg, amoxicillin 1 g, and clarithromycin 500 mg) to treat patients with gastritis and found that compared to patients receiving triple therapy alone (58.6%), those receiving zinc-l-carnosine supplementation (83.33%) achieved a higher eradication rate of H. pylori. The eradication rate of H. pylori in patients of TG (87.27%) post-treatment was markedly inferior to that in CG (63.64%) (P<0.05), consistent with the aforementioned research results. This suggests that the adjunctive use of matrines with omeprazole enteric-coated tablets can effectively increase the eradication rate of H. pylori without increasing toxicity. Furthermore, upon the further comparison of the clinical efficacy among patient groups, it was found that the treatment effectiveness rate in TG (92.73%) was notably superior to that in CG (78.18%) (P<0.05), indicating that the adjunctive use of matrines with omeprazole enteric-coated tablets is more effective than single Western medicine treatment for patients with CAG.

However, due to its single-centre design, the study is subject to certain limitations and selection biases. To address this, future research endeavours will consider enlarging the sample size and including data from multiple hospitals to enhance the representativeness and generalizability of the study findings. Additionally, conducting multi-centre studies and controlling for confounding factors will be undertaken to minimise potential biases and ensure the credibility and scientific rigour of the results. In conclusion, this study can serve as a reference for integrating CAG treatment using both TCM and Western medicine approaches.

## Conclusion

This study retrospectively collected clinical data from 110 patients diagnosed with CAG in the TCM Department of a single hospital. It analysed the clinical efficacy of matrines combined with omeprazole enteric-coated tablets compared to single-agent omeprazole enteric-coated tablets. The results suggest that adjunctive therapy with matrines effectively improves gastric tissue-related symptoms, enhances the eradication rate of H. pylori, and yields more significant clinical efficacy in treating CAG versus singleagent Western medicine therapy.

## Dodatak

### Conflict of interest statement

All the authors declare that they have no conflict of interest in this work.
